# Indirect reference intervals for haematological parameters in capillary blood of pre-school children

**DOI:** 10.11613/BM.2021.010709

**Published:** 2021-02-15

**Authors:** Aleksandra Zeljkovic, Zsófia Csuzdi Balog, Eva Dukai, Jelena Vekic, Zorana Jelic-Ivanovic, Vesna Spasojevic-Kalimanovska

**Affiliations:** 1 Department for Medical Biochemistry, Faculty of Pharmacy, University of Belgrade, Belgrade, Serbia; 2 Department of Laboratory Diagnostics, Healthcare Centre Ada, Ada, Serbia; 3 Department of Laboratory Diagnostics, Healthcare Centre Kanjiža, Kanjiža, Serbia

**Keywords:** reference intervals, haematology, paediatrics, capillary blood, sex differences

## Abstract

**Introduction:**

Indirect estimation of reference intervals (RIs) is straightforward and inexpensive procedure for determination of intra-laboratory RIs. We applied the indirect approach to assess RIs for haematological parameters in capillary blood of pre-school children, using results stored in our laboratory database.

**Materials and methods:**

We extracted data from laboratory information system, for the results obtained by automatic haematology analyser in capillary blood of 154 boys and 146 girls during pre-school medical examination. Data distribution was tested, and logarithmic transformation was applied if needed. Reference intervals were calculated by the nonparametric percentile method.

**Results:**

Reference intervals were calculated for: RBC count (4.2-5.4 x10^12^/L), haemoglobin (114-146 g/L), MCH (25.0-29.4 pg), MCHC (321-368 g/L), RDW-SD (36.1-43.5 fL), WBC count (4.5-12.3 x10^9^/L), neutrophils count (1.7-6.9 x10^9^/L) and percentage (29.0-69.0%), lymphocytes count (1.6-4.4 x10^9^/L) and percentage (21.9-60.7%), PLT (165-459 x10^9^/L), MPV (8.1-11.4 fL) and PDW (9.2-14.4%). Gender specific RIs were calculated for monocytes count (male (M): 0.2-1.6 x10^9^/L; female (F): 0.1-1.4 x10^9^/L) and percentage (M: 2.5-18.3%; F: 1.8-16.7%), haematocrit (M: 0.34-0.42 L/L; F: 0.34-0.43 L/L), MCV (M: 73.4-84.6 fL; F: 75.5-84.2 fL) and RDW (M: 12.1-14.3%; F: 11.7-13.9%), due to observed gender differences in these parameters (P = 0.031, 0.028, 0.020, 0.012 and 0.001; respectively). Estimated RIs markedly varied from the literature based RIs that are used in the laboratory.

**Conclusions:**

Indirect method employed in this study enables straightforward assessment of RIs in pre-school children. Herein derived RIs differed from the literature-based ones, indicating the need for intra-laboratory determination of RIs for specific populations and sample types.

## Introduction

Defining intra-laboratory reference intervals (RIs) for biochemical and haematological parameters is highly important for accurate interpretation of laboratory data. According to the Clinical and Laboratory Standards Institute/International Federation of Clinical Chemistry and Laboratory Medicine (CLSI/IFCC) recommendations, the best procedure for defining RIs is the direct approach, which comprises selection of adequate reference population, sampling, laboratory measurements and statistical analysis of the data (*1*). However, this procedure is time-consuming, expensive and therefore difficult to perform. Consequently, many clinical laboratories opt to use manufacturer-provided or literature-suggested RIs (*2*, *3*).

To the best of our knowledge, a comprehensive survey of procedures that are used to define RIs in laboratory haematology has not been performed in Serbia. Nonetheless, a similar analysis conducted in Australia could provide a general insight into the status of RIs for haematological parameters (*4*). Namely, the authors reported that up to 77% of involved laboratories use RIs adopted from other laboratories, textbooks or scientific papers, instead of determination of their own RIs. The problem with RIs becomes even more complex for paediatric populations, having in mind age variations during childhood, as well as gender differences and ethical issues connected with the collection of samples (*5*). Regarding RIs in haematology, it should be noted that general recommendations comprise the use of venous blood for determination of RIs (*6*), while, in contrast, capillary blood is most frequently used material for the assessment of haematological parameters in children.

An alternative method for determination of RIs is so-called indirect approach, whereby RIs are assessed based on the pre-existing results which are stored in laboratory databases (*7*, *8*). This approach is less demanding, easier to perform and does not include any inconvenience for patients, although it contains several limitations, especially with respect to the selection of an adequate study population (*7*). However, due to above-mentioned challenges in recruiting a required number of healthy children and obtaining sufficient amount of blood, an indirect approach is particularly convenient for establishment of RIs in paediatric population.

In this study, we applied the indirect method for determination of haematological RIs in capillary blood of pre-school children. In addition, we explored gender differences and, where necessary, defined gender specific RIs.

## Materials and methods

### Subjects

For this study, we performed a retrospective analysis of data which were stored in the laboratory information system of the Healthcare Centre Kanjiža, Serbia. We used the results for 18 haematological parameters, which were previously determined in capillary blood samples of 300 pre-school children (154 boys and 146 girls), as a part of routine pre-school testing. The used results were collected from three pre-school generations. Sampling was performed in the following periods: February - April 2015 (105 samples), February – April 2016 (97 samples) and January – March 2017 (98 samples).

All children underwent general medical examination prior to sampling and were free of any acute or chronic local or systemic disease. The entire study was conducted according to the ethical principles established by the Helsinki Declaration. The study protocol was approved by the Ethics Committee of the Healthcare Centre Kanjiža.

### Methods

Blood sampling was performed in accordance with the current CLSI guidelines. In short, 500 μL of capillary blood was taken between 7 and 8 am by fingerstick, using BD Microtainer contact-activated lancet and BD Microtainer blood collection tubes containing K_2_-EDTA (Becton, Dickinson and Company, Franklin Lakes, NJ, USA). The analysed parameters comprised total blood count: white blood cells (WBC) count; WBC differential - neutrophils, monocytes and lymphocytes counts and percentages; red blood cells (RBC) count; concentration of haemoglobin; haematocrit (HCT); RBC indices: mean corpuscular volume (MCV), mean corpuscular haemoglobin (MCH), mean corpuscular haemoglobin concentration (MCHC), red blood cell distribution width (RDW), RDW-standard deviation (RDW-SD); platelets (PLT) count; platelets indices: mean platelet volume (MPV) and platelet distribution width (PDW).

All analyses were performed immediately after blood sampling by Sysmex XP-300 automated haematology analyser (Sysmex Corporation Kobe, Kobe, Japan). Red blood cells, WBC and PLT counts were determined by the direct current detection method with coincidence correction. Populations of cells were separated by automatic discriminators based on complex algorithms. Haematocrit is determined according to the RBC count and volume detection of each individual RBC. Haemoglobin concentration was assessed by the non-cyanide method. All analyses were done simultaneously with three levels (normal, high and low) of quality control samples (Eightcheck-3WP, Sysmex Corporation, Kobe, Japan) in each batch of run.

### Statistical analysis

Normality of data for each of the examined parameters was tested by using the Kolmogorov-Smirnov test. In case of skewed data distribution, log-normal transformation was applied and data distribution was tested again. The analysed data are presented as mean ± standard deviation for normally distributed variables, as geometric mean (confidence interval) for variables with log-normal distribution, or as median (interquartile range) for variables with skewed distribution. Gender differences between the measured values of included haematological parameters were estimated by the Student t-test for normal data distribution, or the Mann-Whitney U test for skewed data distribution.

Determination of RIs by the indirect approach was assessed by using statistical procedures that were recommended by the IFCC Committee on Reference Intervals and Decision Limits and previously described in detail (*7*, *9*, *10*). Data distribution histograms were visually inspected and the Tukey test was used for the identification of outliers. Values that were lower than the lower quartile minus 1.5 x interquartile range, or higher than the upper quartile plus 1.5 x interquartile range were identified as outside values. Far out values were identified if lower than the lower quartile minus 3 x interquartile range, or higher than the upper quartile plus 3 x interquartile range. Following the identification, the outliers were omitted from the database and the entire procedure was repeated until no outliers remained. Reference intervals were calculated by the nonparametric percentile method as 2.5^th^ and 97.5^th^ percentiles with 90% confidence intervals. For skewed variables and parameters that significantly differed between boys and girls, gender-specific RIs were calculated.

All statistical analyses were performed by using MedCalc statistical package (version 12.1.4.0 MedCalc software, Ostend, Belgium). Significant differences are considered for P < 0.05.

## Results

This study included results of haematological parameters determination in capillary blood of 300 pre-school children (median age: 6 year 3 months; min: 5 years 11 months; max: 7 years 1 month). The results of data distribution testing are presented in Table 1. Gaussian distribution was achieved for RBC count, haemoglobin concentration, MCV, MCH, MCHC, percentages of neutrophils, lymphocytes and monocytes, RDW-SD, PLT count and MPV. Normality of data was accomplished after logarithmic transformation for WBC count, neutrophils count, lymphocytes count and PDW. On the other hand, data distribution was skewed for monocytes count, haematocrit and RDW (Table 1). For the establishment of indirect RIs, we visually inspected data distribution histograms with an aim to exclude the outliers. The total numbers of outliers and included data values for RIs estimation for each parameter are presented in Table 1. As it can be seen, the highest number of excluded data is achieved for WBC count and differential, but it was less than 10% in each case.

**Table 1:** Data distribution, number of outliers and total nuber of data included in indirect estimation of reference intervals (RIs)

**Parameter (unit)**	Normality of data*	**Outliers (N)**	**Included data (N)**
RBC (x10^12^/L)	0.473	3	297
WBC (x10^9^/L)	0.321^†^	17	283
Neutrophils (x10^9^/L)	0.480^†^	20	280
Neutrophils (%)	0.433	8	292
Lymphocytes (x10^9^/L)	0.064^†^	24	276
Lymphocytes (%)	0.886	6	294
Monocytes (x10^9^/L)	< 0.001	22	278
Monocytes (%)	0.152	26	274
Haematocrit (L/L)	< 0.001	4	296
Haemoglobin (g/L)	0.264	3	297
MCV (fL)	0.268	2	298
MCH (pg)	0.355	7	293
MCHC (g/L)	0.220	0	300
RDW (%)	< 0.01	6	294
RDW-SD (fL)	0.584	3	297
PLT (x10^9^/L)	0.912	2	298
MPV (fL)	0.121	0	300
PDW (%)	0.084^†^	7	293
*P values obtained by the Kolmogorov-Smirnov test for data normality testing are presented. ^†^Normality of data was achieved following logarithmic transformation. RBC - red blood cells count. WBC - white blood cells count. MCV - mean corpuscular volume. MCH - mean corpuscular haemoglobin. MCHC - mean corpuscular haemoglobin concentration. RDW - red blood cell distribution width. RDW-SD - RDW-standard deviation. PLT - platelets count. MPV - mean platelet volume. PDW - platelet distribution.			

Table 2 represents the values of analysed haematological parameters separately for pre-school boys and girls. Among all tested parameters, statistically significant differences between sexes were achieved for total number and percentage of monocytes, haematocrit, MCV and RDW. The obtained values for the rest of included parameters were comparable between sexes.

**Table 2:** Values of analysed haematological parameters obtained in population of pre-school children according to gender

**Parameter (unit)**	**Boys (N = 154)**	**Girls (N = 146)**	**P**
RBC (x10^12^/L)	4.80 (4.58-5.02)	4.79 (4.59-5.02)	0.731
WBC (x10^9^/L)*	7.70 (6.60-9.10)	7.50 (6.40-9.20)	0.834
Neutrophils (x10^9^/L)*	3.60 (2.80-4.80)	3.70 (2.90-4.90)	0.357
Neutrophils (%)	48.15 (41.00-56.10)	50.05 (41.80-56.30)	0.467
Lymphocytes (x10^9^/L)*	3.10 (2.50-3.60)	3.05 (2.50-3.60)	0.683
Lymphocytes (%)	40.40 (33.50-46.50)	40.20 (34.30-47.50)	0.838
Monocytes (x10^9^/L)^†^	0.80 (0.60-1.10)	0.70 (0.50-0.90)	0.031
Monocytes (%)	10.45 (7.50-13.50)	9.40 (7.40-11.70)	0.028
Haematocrit (L/L)^†^	0.38 (0.37-0.39)	0.38 (0.37-0.40)	0.020
Haemoglobin (g/L)	129.00 (124.00-135.00)	130.00 (126.00-137.00)	0.091
MCV (fL)	79.30 (77.50-81.00)	80.20 (78.30-81.70)	0.012
MCH (pg)	27.00 (26.30-27.90)	27.30 (26.60-28.00)	0.073
MCHC (g/L)	340.00 (334.00-350.00)	341.00 (332.00-350.00)	0.883
RDW (%)^†^	13.00 (12.60-13.50)	12.80 (12.30-13.10)	< 0.001
RDW-SD (fL)	39.80 (38.70-41.10)	39.50 (38.20-41.00)	0.293
PLT (x10^9^/L)	307.50 (259.00-367.00)	308.00 (264.00-351.00)	0.943
MPV (fL)	9.50 (9.00-10.10)	9.50 (8.90-10.10)	0.440
PDW (%)*	11.35 (10.50-12.60)	11.20 (10.40-12.30)	0.240
Data are presented as median (interquartile range) and compared by the Student t-test. *Data are log-transformed prior to analysis and compared by the Student t-test. ^†^Data are compared by the Mann-Whitney U test. RBC - red blood cells count. WBC - white blood cells count. MCV - mean corpuscular volume. MCH - mean corpuscular haemoglobin. MCHC - mean corpuscular haemoglobin concentration. RDW - red blood cell distribution width. RDW-SD - RDW-standard deviation. PLT - platelets count. MPV - mean platelet volume. PDW - platelet distribution.			

In case of haematological parameters which did not differ between sexes, we used the entire dataset for calculations of gender non-specific RIs. The estimated indirect RIs for RBC count, WBC count and PLT-related parameters are presented in Table 3. For each RI, 90% CI for upper and lower reference limits are given.

**Table 3:** Indirectly established gender non-specific reference intervals for haematological parameters

**Parameter (unit)**	**RI**	**90% CI for lower reference limit**	**90% CI for upper reference limit**
RBC (x10^12^/L)	4.2-5.4	4.1-4.3	5.3-5.5
Haemoglobin (g/L)	114-146	113-117	143-147
MCH (pg)	25.0-29.4	24.7-25.2	29.1-29.9
MCHC (g/L)	321-368	319-321	365-372
RDW-SD (fL)	36.1-43.5	35.6-36.7	43.0-43.9
WBC (x10^9^/L)	4.5-12.3	4.3-4.8	11.4-12.6
Neutrophils (x10^9^/L)	1.7-6.9	1.4-1.9	6.3-7.2
Neutrophils (%)	29.0-69.0	26.0-31.0	66.2-72.6
Lymphocytes (x10^9^/L)	1.6-4.4	1.2-1.7	4.2-4.6
Lymphocytes (%)	21.9-60.7	19.7-23.2	58.8-62.4
PLT (x10^9^/L)	165-459	142-178	449-482
MPV (fL)	8.1-11.4	8.0-8.3	11.2-11.5
PDW (%)	9.2-14.4	8.9-9.5	14.3-14.9
RI - reference interval. RBC - red blood cells count. MCH - mean corpuscular hemoglobin. MCHC - mean corpuscular haemoglobin concentration. RDW-SD - RDW-standard deviation. WBC - white blood cells count. PLT - platelets count. MPV - mean platelet volume. PDW - platelet distribution. CI - confidence intervals.			

Due to preliminarily observed differences between boys and girls, gender specific RIs were established for number and percentage of monocytes, haematocrit, MCV and RDW (Table 4). The same, indirect procedure was applied and RIs with 90% CI for upper and lower reference limits are presented.

**Table 4:** Indirectly established gender-specific reference intervals for haematological parameters

**Parameter (unit)**	**Gender**	**RI**	**90% CI for lower reference limit**	**90% CI for upper reference limit**
Monocytes (x10^9^/L)	M	0.2-1.6	0.1-0.3	1.5-1.8
	F	0.1-1.4	0.1-0.2	1.3-1.5
Monocytes (%)	M	2.5-18.3	1.7-4.1	16.8-20.8
	F	1.8-16.7	1.2-2.5	14.0-17.5
Haematocrit (L/L)	M	0.34-0.42	0.34-0.35	0.42-0.42
	F	0.34-0.43	0.33-0.35	0.42-0.43
MCV (fL)	M	73.4-84.6	72.5-74.5	83.6-85.2
	F	75.5-84.2	74.0-75.8	83.6-86.0
RDW (%)	M	12.1-14.3	11.9-12.2	14.0-14.7
	F	11.7-13.9	11.5-12.0	13.8-14.2
RI - reference interval. M – males. F – females. MCV - mean corpuscular volume. RDW - red blood cell distribution width. CI - confidence intervals.				

Finally, we compared the newly calculated RIs with the literature-based RIs that were in use in our Healthcare Centre laboratory’s everyday practice (Table 5). In comparison with the literature-based ones, newly established RIs were shifted towards lower values in case of neutrophils (%), haemoglobin, MCH, RDW-SD and PDW. Higher calculated RIs were observed for WBC, neutrophils and lymphocytes counts, lymphocytes (%), PLT and MPV. Currently estimated indirect RI for RBC was narrower when compared to the literature-based one, while wider in case of MCHC. The observed differences between newly established and currently used RIs ranged from 0 to 47.5%, whilst more prominent differences were observed for upper reference limits. The highest disagreement was recorded in case of gender-specific RIs for total number and percentage of monocytes and haematocrit when compared to gender-nonspecific literature-based RIs.

**Table 5:** Comparison of estimated indirect reference intervals (RIs) from this study with currently used literature-based RIs in everyday laboratory practice

**Parameter (unit)**	**Estimated indirect RIs**	**Literature-based RIs**	*Lower RI diff. (%)	*Upper RI diff. (%)
RBC (x10^12^/L)	4.2-5.4	3.8-5.8	+ 9.7	- 8.2
WBC (x10^9^/L)	4.5-12.3	4.0-10.0	+ 11.1	+ 18.7
Neutrophils (x10^9^/L)	1.7-6.9	1.4-6.5	+ 17.7	+ 5.8
Neutrophils (%)	29.0-69.0	42.2-75.2	- 45.5	- 9.0
Lymphocytes (x10^9^/L)	1.6-4.4	1.2-3.4	+ 25.0	+ 22.7
Lymphocytes (%)	21.9-60.7	20.5-51.1	+ 6.4	+ 15.8
Monocytes (x10^9^/L)	M: 0.2-1.6	0.1-0.84	+ 44.4	+ 47.5
	F: 0.1-1.4		0	+ 40.0
Monocytes (%)	M: 2.5-18.3	2.0-13.0	+ 18.7	+ 28.9
	F: 1.8-16.7		- 11.1	+ 22.2
Haematocrit (L/L)	M: 0.34-0.42	0.35-0.55	- 2.9	- 31.0
	F: 0.34-0.43		- 2.9	- 27.9
Haemoglobin (g/L)	114-146	120-180	- 5.3	- 23.3
MCV (fL)	M: 73.4-84.6	80.0-99.9	- 9.0	- 18.2
	F: 75.5-84.2		- 6.0	- 18.7
MCH (pg)	25.0-29.4	27.0-32.0	- 8.0	- 8.8
MCHC (g/L)	321-368	330-360	- 2.8	+ 2.2
RDW (%)	M: 12.1-14.3	11.0-16.0	+ 8.9	- 11.7
	F: 11.7-13.9		+ 6.0	- 14.8
RDW-SD (fL)	36.1-43.5	37.0-54.0	- 2.5	- 24.1
PLT (x10^9^/L)	165-459	150-450	+ 9.1	+ 2.0
MPV (fL)	8.1-11.4	6.0-11.0	+ 25.9	+ 3.5
PDW (%)	9.2-14.4	10.0-16.0	- 8.7	- 11.1
*Values represent differences between indirectly estimated RIs and literature-based RIs, expressed as percentages. Directions of changes are represented by (+) if indirectly estimated RIs are higher than literature-based ones, or by (-) if indirectly estimated RIs are lower than literature-based ones. M – males. F – females. RBC - red blood cells count. WBC - white blood cells count. MCV - mean corpuscular volume. MCH - mean corpuscular haemoglobin. MCHC - mean corpuscular haemoglobin concentration. RDW - red blood cell distribution width. RDW-SD - RDW-standard deviation. PLT - platelets count. MPV - mean platelet volume. PDW - platelet distribution.				

## Discussion

In this study, we used the indirect method for the assessment of intra-laboratory RIs for 18 haematological parameters in pre-school children. Unique RIs were calculated for those parameters that did not differ between sexes, while gender-specific RIs were derived in case of parameters that significantly varied among boys and girls. Defining internal laboratory RIs is a demanding procedure in terms of both time and resources. Thus, many routine laboratories preferably use externally derived RIs, although such approach might be associated with errors and misinterpretation of the obtained laboratory results (*4*, *11*, *12*). Recommendations of CLSI and IFCC strongly support development of internal RIs and more recently, an easier and less consumable indirect method was proposed (*7*). In this study, we used the indirect method for the assessment of haematological RIs in pre-school children. This method offers several advantages over the traditional direct approach, which are mainly related to minimized use of laboratory resources. First of all, indirect method comprises the usage of pre-existing datasets stored in laboratory storage systems, thus time-consuming recruitment of eligible subjects, collecting and analysis of samples are avoided. Consequently, financial costs and engagement of laboratory staff are minimized. It is also important to mention that same preanalytical and analytical conditions that are routinely used in everyday laboratory practice are as well applied to determination of RIs. Moreover, patients whose results will be interpreted by using indirect RIs belong to the very same general population that is used for RIs calculation. It is also noteworthy that the usage of available dataset ensures adequate number of samples of different age, gender and ethnicity (*7*).

The main source of errors during the indirect assessment of RIs is related to the selection of an adequate database. The principal goal in this process is to reduce potential inclusion of samples affected by any disease, or a condition that could influence definition of RIs. Herein we used the results obtained from healthy outpatients who were routinely tested during pre-school medical examinations. The advantages of using the results obtained from apparently healthy subjects are clear, since such approach warrants less interferences related to the presence of inflammation, various diseases, medical treatments, dietary changes and other alterations that are frequently seen in in-hospital patients (*7*). In this study, prevalence of outliers was low for any of analysed parameters, thus implying relative homogeneity of datasets and its adequacy for the assessment of reliable RIs.

Next important issue for the estimation of RIs is sample size. It has been suggested that minimal number of samples for estimation of RIs should be 400 (*9*). However, it is also stated that for specific homogenous age groups even smaller sample size could provide adequate results (*7*). For this study purposes, we used data which belongs to very homogenous age group, thus smaller sample size is adequate for calculation of RIs. In addition, according to the Canadian laboratory initiative on pediatric reference intervals (CALIPER), the minimal sample size for establishing paediatric RIs is 120, and this prerequisite was accomplished in our study, even when gender-specific RIs are calculated (*13*, *14*).

Another significant question for the statistical approach in the assessment of RIs is data distribution. Namely, general assumption of the recommended statistical procedures for calculation of RIs is that the analysed data set is normally distributed (*15*, *16*). As it has been recently suggested, any data set with skewed distribution should be analysed for subpopulations, in terms of gender, age and other criteria (*7*, *17*). Applying of these suggestions in our study led us to data partitioning by gender and determination of gender-specific RIs. Although the distribution of MCV and monocytes percentage was not skewed, the rationale for gender partitioning lies in the finding that these parameters significantly differ between sexes as well. No partitioning by age was necessary since our study population was homogenous by age. Except for monocytes, all other parameters that differed between boys and girls were related to RBC indices. Gender differences in haemoglobin level ad RBC indices are well known in adult populations, but these variations are not sufficiently explored in children (*18*). Recently, a study of age and sex differences in RBC variables was conducted in a population of Macedonian children aged 8-18 years (*19*). The authors reported higher values of RBC variables in boys, which is in contrast to our results. On the other hand, a study performed in Tanzanian children aged 13-18 years showed higher RBC count, haemoglobin and haematocrit, but lower MCV in boys when compared to girls (*20*). However, our study participants were younger than those included in the above-mentioned studies, suggesting that differences in haematological markers in children should be further considered with respect to gender and age, but also ethnicity (*19*, *20*). The latest report from the CALIPER study enrolling 536 children and adolescents of multi-ethnic origin aged 0-21 years has shown that neither haematocrit, MCV or RDW values differed between sexes in the age group correspondent to our study. Although, monocyte percentage was higher in boys than in girls, such increase was significant only in subjects older than 15 years (*21*). Yet, Aldrimer *et al.* in a study of paediatric population of Caucasian origin pointed towards higher MCV values in girls starting at age of 4, which is consistent with our findings (*22*). Interestingly, a study of age- and sex-related dynamics in biochemical and haematological parameters in large German paediatric cohort indicated that, although significant differences were not recorded, a trend of slightly higher MCV values in girls and RDW values in boys aged 6-8 years was observed, similarly as in our research (*23*). Taken altogether, the issue of age and gender specificities in paediatric haematology parameters is complex and requires further investigations.

An important aspect of our study is that RIs were determined based on samples of capillary blood. It has been shown that variations in haematological markers are present in children and adults depending whether venous or capillary blood is used (*24*-*26*). However, haematological RIs in paediatrics are often determined in venous blood samples, even it is well known that capillary blood is more frequently used, especially in primary healthcare centres. Herein we used data derived from capillary blood samples, thereby making our defined RIs even more applicable in routine paediatric healthcare practice. By using capillary blood data sets we ensured the assessment of RIs that better suits for the specific needs of this particular laboratory.

To get a better insight into the significance of intra-laboratory developed RIs, we compared the RIs determined in this study with the literature-based ones that are currently in use in our laboratory. Although the estimated deviations between the two categories of RIs ranged 0-20% for majority of cases, our analysis indicated some major differences that went up to 47.5%. Such findings emphasize the need for development of intra-laboratory RIs, in order to minimize the possibility of diagnostic errors due to inadequate reference ranges. The observed discrepancies might arise due to the fact that literature-based RIs were defined for venous blood samples. Thus, the development of intra-laboratory RIs, which are adapted to specific laboratory needs and types of samples, is an optimal approach for improving this aspect of laboratory diagnostics. Moreover, literature-based RIs did not recognize gender-specific variations in several parameters, which we identified as significantly different among sexes. Interestingly, the highest level of disagreement between estimated and literature-based RIs was observed for those parameters that differed between boys and girls, thus confirming the inappropriateness of gender-nonspecific RIs in these cases.

It should be mentioned that RIs in this study are determined by using 3-part differential haematology analyser, so only three WBC populations are evaluated. For more comprehensive determination of haematological RIs, 5-part differential haematology analyser should be employed.

In conclusion, by using the relatively simple indirect method we obtained adequate intra-laboratory reference ranges for haematological parameters in pre-school children. For parameters that significantly differ among sexes, separate RIs for boys and girls are needed. The observed differences among herein-calculated and literature-based RIs accentuate the need for intra-laboratory estimation of RIs, with respect to the sample type and targeted population. Accurate and reliable RIs for haematology analytes in children of different age and gender are of great significance for diagnostics and monitoring of therapy in this specific population.
